# A grass-specific structural myosin VIII regulates protoxylem development and hydraulic conductance in sorghum

**DOI:** 10.1093/plphys/kiag712

**Published:** 2026-09-22

**Authors:** Zhiyuan Liu, Ran Tian, Leonidas D’Agostino, Zhanguo Xin, Junping Chen, Gunvant Baliram Patil, Yinping Jiao

**Affiliations:** Department of Plant and Soil Science, Institute of Genomics for Crop Abiotic Stress Tolerance, Texas Tech University, 2500 Broadway, Lubbock, TX 79409, United States; Department of Plant and Soil Science, Institute of Genomics for Crop Abiotic Stress Tolerance, Texas Tech University, 2500 Broadway, Lubbock, TX 79409, United States; Department of Plant and Soil Science, Institute of Genomics for Crop Abiotic Stress Tolerance, Texas Tech University, 2500 Broadway, Lubbock, TX 79409, United States; USDA-ARS, Cropping Systems Research Laboratory, Plant Stress and Germplasm Development Unit, 3810 4th Street, Lubbock, TX 79415, United States; USDA-ARS, Cropping Systems Research Laboratory, Plant Stress and Germplasm Development Unit, 3810 4th Street, Lubbock, TX 79415, United States; Department of Plant and Soil Science, Institute of Genomics for Crop Abiotic Stress Tolerance, Texas Tech University, 2500 Broadway, Lubbock, TX 79409, United States; Department of Plant and Soil Science, Institute of Genomics for Crop Abiotic Stress Tolerance, Texas Tech University, 2500 Broadway, Lubbock, TX 79409, United States

## Abstract

Sorghum (*Sorghum bicolor*), a C_4_ grass adapted to hot semi-arid environments, depends on reinforced xylem vessels to maintain hydraulic conductance under high evaporative demand. During xylem differentiation, coordinated microtubule and actin dynamics guide secondary cell wall (SCW) deposition; however, whether actin-based motors regulate vascular architecture and hydraulic performance has remained unknown. Here, we identify HEAT-SENSITIVE 1 (HS1), a previously uncharacterized myosin VIII, as a central regulator of protoxylem (PX) integrity and water transport in sorghum. The hs1 mutant exhibited severe leaf scorching under field conditions, accompanied by pronounced PX defects under controlled environments, including vessel collapse, reduced lumen area, and attenuated lignified SCWs. These structural abnormalities compromised longitudinal hydraulic conductance, diminished whole-plant water use, and rendered developing leaves unable to meet transpirational demand, resulting in transient water deficit and secondary tissue injury. HS1 encoded a grass-specific myosin VIII with a grass-specific N-terminal extension exhibiting high intrinsic disorder in N-terminal and lineage-specific substitutions in the motor domain. Single-cell transcriptome analyses positioned HS1 within differentiating PX cells of developing leaves, revealing pronounced temporal and cell type specificity. Furthermore, bulk transcriptome profiling and quantitative lignin measurements indicated that HS1 is required for proper lignin deposition during PX differentiation, linking an actin-based motor to wall reinforcement. Reduced nucleotide diversity at the HS1 locus further supports strong evolutionary constraint, suggesting an adaptive role in sorghum hydraulic resilience. Together, these findings establish HS1 as an actin-based motor controlling xylem architecture and hydraulic function in plants, a previously unrecognized component of grass hydraulic regulation not evident in Arabidopsis.

## Introduction

Sorghum is a diploid C_4_ grass of the Poaceae family, the fifth most produced cereal crop worldwide (Fontanet-Manzaneque et al. 2025; Nigam et al. 2025; Zhang et al. 2025). Among the major grass crops, such as wheat, rice, maize, and barley, sorghum exhibits outstanding heat and drought tolerance, making it a critically important crop for agricultural production in arid and semi-arid regions (Parent and Tardieu 2012; Gidi 2023). Understanding the mechanistic basis of this resilience has become crucial under increasingly adverse environmental conditions in crop production.

The highly efficient xylem hydraulic system is a key contributor to sorghum's tolerance to heat and drought stress. This efficiency is largely determined by xylem anatomical features such as vessel diameter and secondary cell wall (SCW) thickness, which support water transport efficiency, limit cavitation, and sustain transpiration (Drobnitch et al. 2021; Lehrer and Hawkins 2023; Torres-Ruiz et al. 2024). Hydraulic efficiency ultimately depends on the coordinated development of xylem cell types with lignified SCWs, which provide mechanical strength, structural integrity, and limit cavitation (Rogers and Campbell 2004; Hwang et al. 2016; Chen et al. 2025). Among these, protoxylem (PX) represents the earliest differentiated xylem tissue, initiates water transport during early organ development, and is particularly prone to collapse during tissue elongation (Goodwin 1942; Sharman 1942). By contrast, metaxylem (MX) vessels complete differentiation only after surrounding tissues have finished elongation and subsequently serve as the principal conduits for water movement in mature organs, such as mature leaves (Goodwin 1942; Martre et al. 2000; Huang et al. 2024). Xylem development is orchestrated through multiple, tightly coordinated regulatory layers that operate across distinct developmental stages. Xylem development is governed by complex and highly integrated regulatory networks. These include hierarchical transcriptional programs driven by NAC master regulators such as VND7 and SND1 (Kubo et al. 2005; Yamaguchi and Demura 2010) and their downstream MYB factors, including MYB46 (Zhong et al. 2007; Xiao et al. 2021), coordinated activation of enzymes responsible for SCW biosynthesis (Turner and Somerville 1997; Taylor et al. 2004; Wu et al. 2009), tightly regulated hydrolase activities that execute programmed cell death to generate functional conduits (Groover and Jones 1999; Funk et al. 2002), and cytoskeletal dynamics spatially regulate SCW deposition (Oda and Fukuda 2013; Higa et al. 2024). Among cytoskeletal regulators, motor proteins, including kinesins and myosins, represent a key but underexplored layer (Nebenführ and Dixit 2018; Ryan and Nebenführ 2018). To date, only 2 kinesins have been functionally correlated in xylem development: Kinesin-13A, which regulates MX pit size, and FRAGILE FIBER1 (FRA1), which controls PX SCW thickness through microtubule organization (Oda and Fukuda 2013; Zhu et al. 2015). Both genes modulate microtubule stability and organization during SCW formation in *Arabidopsis*. Despite these roles in xylem patterning, direct evidence linking kinesin activity to hydraulic function is lacking, and no myosin has yet been functionally characterized for xylem development or water transport.

Myosins are evolutionarily conserved actin-based motor proteins that drive intracellular transport and spatial organization of cellular components (Madison and Nebenführ 2013). In plants, the myosin superfamily comprises 2 major classes, myosin XI and myosin VIII, which differ in abundance, localization, and biological function (Peremyslov et al. 2011; Ryan and Nebenführ 2018). Myosin XI proteins have been shown to contribute to stem proprioception in *Arabidopsis*, protein body formation during maize endosperm development, and powdery mildew resistance in barley (Wang et al. 2012; Okamoto et al. 2015; Acevedo-Garcia et al. 2022). To date, functional characterization of myosin VIII genes remains limited. Studies in moss have linked myosin VIII to cell division and developmental patterning (Wu et al. 2011; Wu and Bezanilla 2014), while in *Arabidopsis*, several myosin VIII isoforms facilitate *Agrobacterium*-mediated transformation (Liu et al. 2026). *Arabidopsis thaliana* MYOSIN 1 (ATM1; AT3G19960) is the only myosin VIII member shown to regulate a developmental function, controlling root meristem cell proliferation via auxin and sugar signaling pathways (Olatunji et al. 2023). To date, few myosin VIII genes have been functionally linked to a defined plant trait at the single-gene level. We hypothesize that HS1, a grass-specific class VIII myosin gene, is a key regulator of PX integrity and water transport in sorghum, functioning through the modulation of lignin deposition during SCW formation. Our findings reveal a previously unrecognized function of myosin in grass vascular development and establish HS1 as a critical regulator of xylem structure and hydraulic performance.

## Materials and methods

### Identification of the causal mutation of *hs1*

The *hs1* mutant was identified through field screening of pedigreed M_3_ populations derived from EMS-treated BTx623 (Jiao et al. 2016) at the USDA research fields in Lubbock, Texas. Field trials were conducted under fully irrigated conditions using a subsurface drip irrigation system. To map the causal mutation, the *hs1* mutant was crossed with BTx623 to generate F_1_ plants, and F_2_ populations were evaluated for the leaf scorching phenotype under field conditions during the 2018 and 2021 growing seasons. Identification of the causal mutation using bulked segregant analysis (BSA) and whole-genome resequencing is described in Methods S1.

### Physiological characterization of *hs1* under a controlled environment

For all controlled-environment experiments, wild type (WT) and *hs1* sorghum plants were cultivated in a greenhouse (Lubbock, TX, USA) under standard irrigation. Plants were maintained at 28/25 °C (day/night) with a 16 h light/8 h dark photoperiod. Due to diurnal fluctuations, the actual greenhouse temperature ranged from approximately 26 to 30 °C, within the optimal range for sorghum growth. All experiments below were conducted at the growing point differentiation (GPD) stage (∼30 d after planting).

For root assays, root systems were removed from the soil, washed, and imaged for morphological analysis, and xylem sap exudation was measured from cut stems. For stomatal analysis, epidermal impressions were obtained from developing leaves and used to quantify stomatal density and size. For water transport assays, the excised whole above-ground portion or leaves were placed in safranin solution to monitor dye uptake. For leaf water-loss assays, detached leaves were weighed over time to determine water loss. Leaf physiological parameters, including H_2_O_2_ accumulation, relative water content (RWC), chlorophyll content, and electrolyte leakage (EL), were also measured. Further details are provided in Methods S2.

### Scanning electron microscopy

Fresh midribs from developing leaf bases of WT and *hs1* plants were collected before visible symptoms appeared and at night, when transpiration was low, to minimize the possibility of secondary xylem collapse caused by high transpiration. Approximately 2-mm transverse sections were excised, immediately frozen in liquid nitrogen, and lyophilized overnight (Model 117, Labconco). Dried sections were either kept transverse or further cut longitudinally, mounted on aluminum stubs, and imaged using a Hitachi S-3400 scanning electron microscope at 20 keV. Lumen area, long axis, short axis, and SCW thickness of PX vessels were measured manually in ImageJ (Schneider et al. 2012). All PX vessels in the midrib were measured, with 3 biological replicates per genotype.

### Transcriptome profiling of the leaf base midrib

WT and *hs1* plants were grown in the greenhouse under the optimal conditions described above. At the GPD stage (∼30 d after planting), samples were collected in the early morning, before the appearance of visible phenotypic symptoms, when transpirational demand was low. Total RNA was extracted from the midrib base of both mature leaves and developing leaves (the leaf just above the last fully expanded mature leaf) using the RNeasy Plant Mini Kit (74904; QIAGEN) according to the manufacturer's instructions. RNA sequencing libraries were prepared using standard Illumina protocols and sequenced by a commercial service provider. For each genotype and tissue type, 3 independent biological replicates were sequenced. RNA-seq data processing and differential expression analyses were performed as previously described (Jiao et al. 2018) with minor modifications, primarily the use of the updated *Sorghum bicolor* BTx623 reference genome (version 3). Further details are provided in Methods S3.

### Identification of orthologs

Homologs of xylem development genes characterized in *A. thaliana*, *Zea mays*, and *Oryza sativa* were identified in sorghum using BLAST searches against the *Sorghum bicolor* BTx623 genome (v3.1). Candidate homologs were defined as hits with ≥30% sequence similarity, *E*-value ≤1e−5, and score ≥100 among the top 5 matches. Reciprocal BLAST searches were then performed using these top hits against the sorghum protein sequences, and orthology was inferred when the original query was recovered among the top 3 hits.

### Phylogenetic analysis

Two phylogenetic analyses were performed to classify HS1 as myosin VIII or XI and to determine its phylogenetic position within the myosin VIII clade. For the classification analysis, myosin VIII and XI sequences from Arabidopsis, maize, rice, and sorghum were collected from published datasets and genomic databases (Reddy and Day 2001a; Goodstein et al. 2012; Kawahara et al. 2013; Sakai et al. 2013; Wang et al. 2014). For the clade-level phylogenetic analysis of myosin VIII, 134 myosin VIII homologs from 44 land plant species were retrieved from Ensembl Plants via BioMart (Kinsella et al. 2011; Yates et al. 2022). Detailed procedures for sequence alignment and phylogenetic inference are provided in Methods S4.

### Terminal feature analysis of myosin VIII proteins

Protein domain architecture was determined using InterProScan (Jones et al. 2014) with ProSiteProfiles annotations (Sigrist et al. 2026). The N-terminal region was defined as the sequence preceding the first detected SH3 domain, while the C-terminal boundary was defined by the position of the last detected IQ motif. N-terminal sequence motifs were analyzed using MEME (Bailey et al. 2015) and visualized with TBtools (Chen et al. 2020). Intrinsic disorder (IDR) of myosin VIII proteins was predicted using IUPred2A (Mészáros et al. 2018) in long-disorder mode. N-terminal IDR length was defined as residues with IUPred2 scores ≥0.5. For visualization, normalized residue positions were mapped onto a representative reference protein (HS1). Detailed procedures for IUPred2-based disorder profiling are provided in Methods S5.

### Motor domain feature analysis of myosin VIII proteins

Motor domain boundaries were defined using Pfam annotations, and sequences were aligned with hmmalign in HMMER v3.1b2 (Eddy 2011). Functional regions, including the purine-binding loop, P-loop, switch I, switch II, relay loop, and a downstream converter-region window, were defined based on residue positions in a representative reference protein (HS1). Corresponding alignment blocks were extracted from the alignment and verified manually. Feature-specific alignments were visualized in Jalview (Waterhouse et al. 2009), and representative sequences from each phylogenetic group were selected for display in the main figures. Sequence logos were generated to summarize residue conservation.

### ATP docking analysis

ATP docking analysis was performed using ATPdock (Rao et al. 2022). First, ATP binding sites for previously generated PDB files (see Protein structure and stability analysis**)** were obtained using ATPbind (Hu et al. 2018). PDB files and binding site information were analyzed using ATPdock with default parameters to estimate the binding free energy for each protein. ATP pocket visualization was performed with PyMOL (Schrodinger 2015).

### Single-cell RNAseq analysis

The publicly available single-cell transcriptomics data from Swift et al. (2024) were obtained from the NCBI Gene Expression Omnibus (GSE248919) for further analysis. Single-cell raw reads from D’Agostino et al. (2025) were obtained from the authors and processed as detailed in the manuscript. Analysis was performed in R using the Seurat package (version 4.4) (Hao et al. 2021). Cells with no assigned cell type were removed before further investigation. Xylem cells were subset from the whole dataset and grouped into 2 groups based on *HS1* gene expression. Cells with >0 normalized expression of *HS1* were labeled as “myosin,” and cells with ≤0 were labeled as “normal.” Gene expression was visualized using the dotplot function in the Seurat package.

### Histological analysis

Midrib samples for lignin staining were collected using the same sampling strategy described above for SEM analysis. Transverse cryosections were then subjected to phloroglucinol–HCl staining (Wiesner reaction) following the method described by Li et al. (2023) with minor modifications. Staining intensity was quantified using ImageJ (Schneider et al. 2012). This quantification strategy was adapted from previous lignin intensity analyses with modifications (Baldacci-Cresp et al. 2020). Detailed procedures are provided in Methods S6.

### Nucleotide diversity analysis

To investigate evolutionary patterns of myosin genes across species, nucleotide diversity (*π*) was calculated at the *HS1* locus in sorghum and its maize ortholog *ZmVIII-3* (*Zm00001d021785*), including the 20 kb flanking regions. Detailed procedures are described in Methods S7.

## Results

### Sorghum heat-sensitive mutant *hs1*

A heat-sensitive sorghum mutant, *hs1*, was isolated from an EMS-induced mutant population generated in the genetic background of reference line BTx623 (Xin et al. 2008; Jiao et al. 2016; Jiao et al. 2024). The mutant was initially designated *heat sensitive 1* based on the consistent appearance of leaf scorching of developing leaves after exposure to a moderate heat stress event (>34 °C) under well-watered field conditions at or after 5-leaf stages and throughout the vegetative developmental stages (Fig. 1a; Fig. S1a to c). The scorching phenotype was initiated at the top part of the developing leaf and moved downward to the mid to lower sections of the leaf. The severity of leaf phenotypes and the number of leaves being affected were correlated to the intensity and duration of a particular heat wave (Fig. S1d). Therefore, multiple leaves showed a leaf-scorching phenotype after multiple heat stress events during the growing season (Fig. 1a; Fig. S1a to c). As a result of heat-induced permanent leaf damage, *hs1* showed significant reductions in leaf length and plant height compared to WT grown under the same environments (*P* < 0.05; Fig. 1b and c; Fig. S1e to h). In addition, the reproductive tissues of *hs1* are also sensitive to heat stress. Heat stress at early stages of panicle development reduced panicle length, at the half bloom stage caused panicle blast (Fig. 1d) and at the pollination stage significantly decreased seed numbers (*P* < 0.01; Fig. 1e and f).

**Figure 1:**
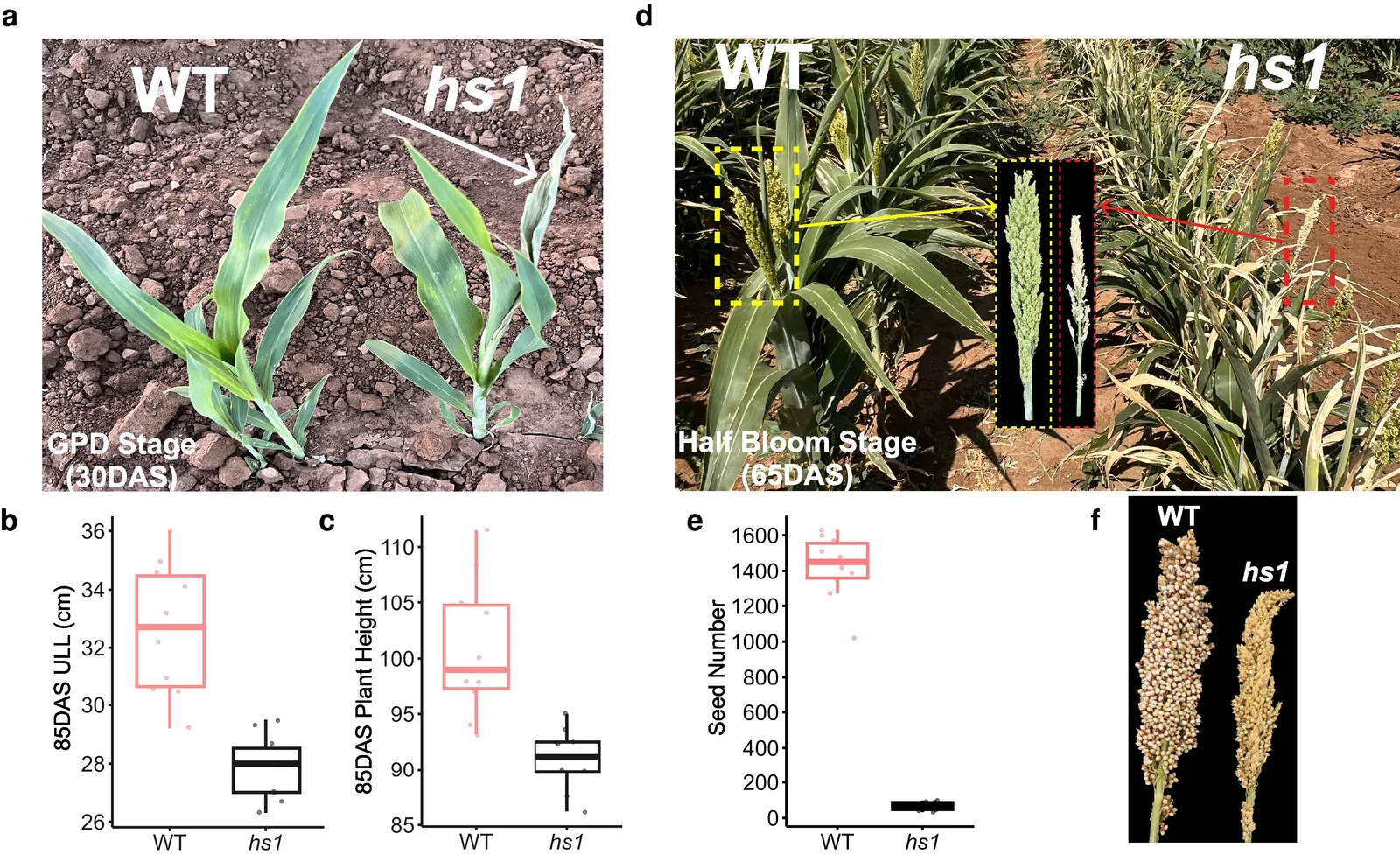
Phenotype of heat-sensitive mutant *hs1*. a) Leaf phenotype of WT (BTx623) and *hs1* grown in a field (Lubbock, TX) at the GPD stage (30 d after sowing, DAS). The white arrow indicates a scorched developing leaf. b, c) Quantification of upper leaf length (ULL) (b) and plant height (c) at 85 DAS. d) Panicle phenotype of WT and *hs1* at the half-bloom stage; the middle panel shows a close-up view. e, f) Quantification (e) and representative images (f) of the seed number at physiological maturity. For statistical analyses, boxes indicate the first and third quartiles, and horizontal lines indicate the median (*n* = 10). **P < 0.01 (2-tailed Student's *t*-test).

Another phenotype in the field related to *hs1* was leaf roll of developing leaves in the afternoon under low relative humidity (RH; 29% to 33%). The rolled leaf partially recovered the next morning but reappeared in the hot afternoons (Fig. S2a). Over time, the rolled leaf became bleached gray in color, leading to permanent leaf damage after repeated daily heat exposure (Fig. S2a). Notably, under greenhouse conditions leaf scorching was also observed in developing *hs1* leaves when transpiration increased at afternoon (12:00 PM), but not in the morning (8:00 AM) and evening (7:00 PM) (Fig. S2b), although plants were maintained under optimal growth conditions for sorghum [defined as 26 °C to 30 °C with natural light ranging from 430 to 1,162 μmol m^−2^ s^−1^ photosynthetic photon flux density {PPFD}]. This diurnal pattern indicates that the visible leaf-scorching phenotype was transient.

### Collapsed PX vessels in developing leaves of *hs1* disrupted water transport

To determine the developmental defects underlying the scorching leaf phenotype, we first examined root traits and stomatal characteristics, which are key components of water uptake and transpiration, under optimal greenhouse conditions in the morning. At the GPD stage, root surface area and root sap (defined as root xylem sap exudate) did not differ significantly between WT and *hs1* (*P* > 0.05; Fig. S3a to d). Similarly, no differences were observed in stomatal size (measured as guard cell length) and density in developing leaves between the 2 genotypes (*P* > 0.05; Fig. S4a to d). Because these traits have been used to assess water acquisition and loss (Li et al. 2023; Huang et al. 2024), our results suggest that root water uptake capacity and stomatal traits were unlikely to be the primary cause of the *hs1* scorching phenotype.

In contrast, safranin uptake assay using cut stems revealed clear differences in water transport between WT and *hs1*. The volume of solutions absorbed by WT was significantly greater than that by *hs1* (*P* < 0.05; Fig. S5a and b). Although mature leaves of both genotypes exhibited similar staining, developing *hs1* leaves showed markedly reduced or absent dye accumulation (Fig. S4c to d). When developing leaves were detached from the whole plant for analysis, dye uptake and midrib transport distance were reduced by 23.1% and 27.3%, respectively, in *hs1* relative to WT (*P* < 0.05; Fig. 2a to c). In *hs1*, staining stopped at the upper midrib, whereas WT leaves showed continuous, more intense staining along the veins (Fig. 2d). Together with previous use of dye-tracing assays to assess hydraulic transport (Li et al. 2023; Huang et al. 2024), these results demonstrate that the leaf-scorching phenotype of *hs1* arises from impaired internal water transport and its distribution in developing leaves, rather than defects in water uptake or stomatal regulation.

**Figure 2:**
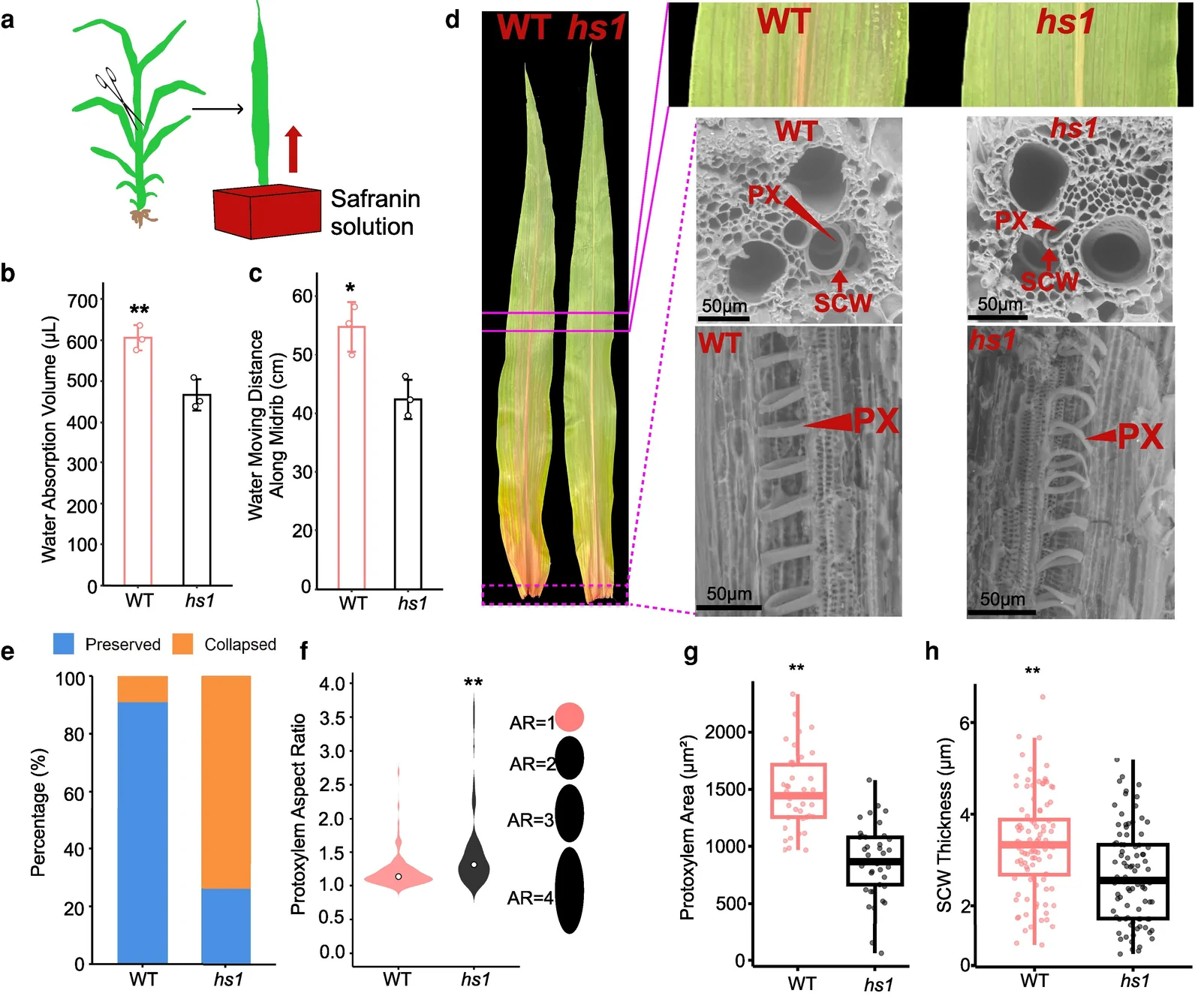
Impaired water transport in developing leaves of *hs1*. a) Schematic of the safranin uptake assay used to assess xylem-mediated water transport. b, c) Quantification of safranin dye uptake (b) and midrib transport distance (c) in WT and *hs1*. Values represent mean ± SD (*n* = 3 biological replicates per genotype). d) Representative developing leaves of WT and *hs1* following the safranin assay. Upper right, enlarged view of leaves. Middle and lower right, scanning electron microscopy (SEM) images of midrib transverse and longitudinal sections from the developing leaf base. PX, protoxylem. Bars = 50 µm. Red triangles mark PX vessels, and red arrows indicate their SCWs. e−h) Quantification of PX integrity in midrib transverse sections from the leaf base of developing WT and *hs1* plants, including frequency of collapsed versus preserved (intact) vessels (e), aspect ratio (f), PX area (g), and SCW thickness (h). Data were obtained from 3 biological replicates per genotype. For each genotype, all measured individual PX vessels from the 3 biological replicates were pooled, and statistical tests were performed on these pooled vessel-level measurements (*n* ≥ 42 for e; *n* ≥ 93 for f; *n* = 41 for g; *n* ≥ 90 for H), rather than on biological replicate means. Boxes represent the first and third quartiles, and horizontal lines indicate medians. Statistical significance was determined using 2-tailed Student's *t*-tests (*P < 0.05; **P < 0.01).

As PX mediates water transport in developing leaves, particularly in the elongating basal region (Goodwin 1942), we examined midrib PX structure at the developing leaf base. In WT, PX vessels were intact and nearly circular, with SCWs confined to the vessel boundary (Fig. 2d). By contrast, *hs1* PX showed a continuum of structural defects, including mild elliptical deformation, spindle-shaped distortion, and SCW extrusion. In severe cases, disintegrated PX remnants accumulated within the vessel cavity (Fig. 2d; Fig. S6a to d). Longitudinal sections showed regularly spaced annular SCW thickenings in WT, whereas those in *hs1* were distorted and irregularly arranged (Fig. 2d). Quantitative analysis confirmed extensive structural defects in *hs1*. Approximately 73.8% of PX vessels were collapsed in *hs1*, compared with 11.11% in WT (Fig. 2e). *hs1* vessels had significantly higher aspect ratios (*P* < 0.01; Fig. 2f), smaller lumen areas (870 ± 310 μm^2^ vs. 1,479 ± 332 μm^2^ in WT; *P* < 0.01; Fig. 2g), and thinner secondary walls (2.66 ± 1.02 µm vs. 3.34 ± 1.05 µm in WT; *P* < 0.01; Fig. 2h).

To determine whether this vascular defect persists across developmental stages under optimal growth conditions, we next examined successive developing leaves. Leaf scorching was consistently observed in *hs1* across successive developmental stages under optimal growth conditions, including the sixth leaf at the 5-leaf stage, the seventh through 11th leaves at the GPD stage, and the flag leaf at the flag-leaf stage (Fig. S7a). Detailed observation of these developing leaves (excluding the ninth, which was shown in Fig. 2e) revealed the presence of collapsed PX at each stage (Fig. S7a). Quantification revealed that the ratios of collapsed xylem in *hs1* ranged from 56% to 74%, which were higher than those in the WT (Fig. S7b). Collectively, these defects compromise PX integrity and provide a structural explanation for reduced water transport in developing *hs1* leaves.

### Physiological consequences of PX collapse

We next evaluated the physiological consequences of PX disruption of leaf water relations in the greenhouse under optimal conditions. During the morning, developing leaves of WT and *hs1* were visually indistinguishable and showed comparable H_2_O_2_ accumulation (Fig. 3a and b). In contrast, in the afternoon, a period typically characterized by higher evaporative demand, the *hs1* developing leaves showed visible scorching and stronger DAB staining compared with WT (Fig. 3c and d). Consistent with these observations, *hs1* leaves’ RWC, chlorophyll concentration (Chl), and EL were indistinguishable from WT in the morning (*P* > 0.05), whereas in the afternoon, *hs1* leaves exhibited significantly reduced RWC and Chl and increased EL (*P* < 0.05; Fig. 3e to g). These changes were largely transient, as approximately 73% of scorched *hs1* leaves recovered greenness after 2 d, whereas WT leaves remained unaffected (Fig. S8). At the whole-plant level, *hs1* plants exhibited significantly lower daily water uptake and reduced stature compared with WT (all *P* < 0.01; Fig. S9a−c). Detached leaf assays further showed slower initial water loss in *hs1* (Fig. S9d to f), consistent with impaired hydraulic conductance. In a separate high-temperature assay, visible leaf scorching was also observed in developing leaves of hs1 plants, further supporting impaired xylem function and reduced water supply under high evaporative demand conditions (Fig. S10). Together, these results demonstrated that the collapse of PX vessels in developing *hs1* leaves compromises vascular integrity and reduces hydraulic efficiency. Consequently, *hs1* leaves experienced transient water deficit, oxidative stress, and leaf scorching, establishing PX integrity as a critical determinant of water transport during leaf development.

**Figure 3:**
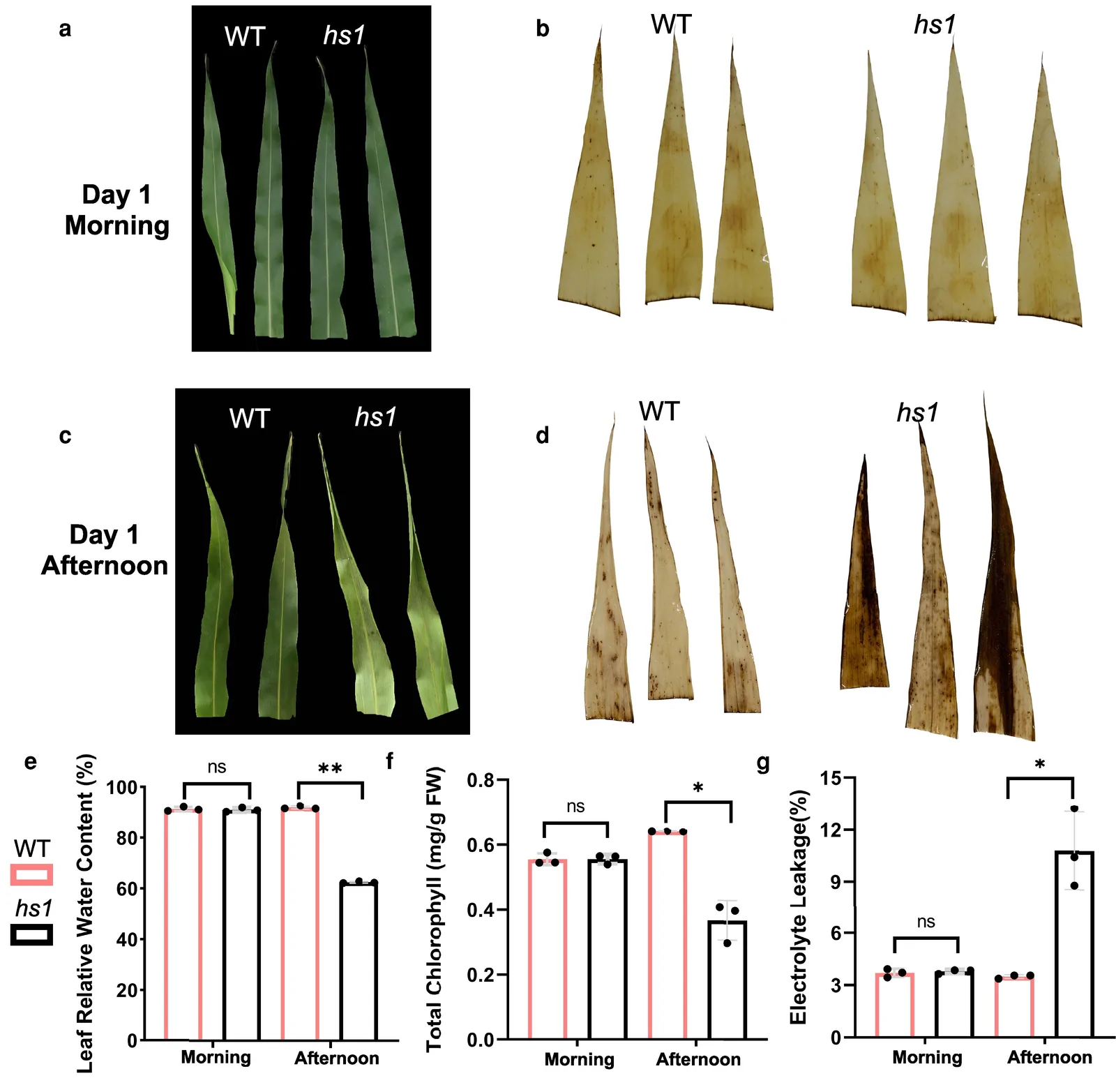
Morphological and physiological characterization of developing leaves in WT and *hs1*. a, b) Developing leaf morphology (a) and 3,3′-diaminobenzidine (DAB) staining (b) in WT and *hs1* plants in the morning (10 AM, CT). c, d) Developing leaf morphology (c) and DAB staining (d) in WT and *hs1* plants in the afternoon (4:30 PM, CT). e−g) Statistical comparisons of leaf relative water content (e), total chlorophyll content (f), and electrolyte leakage (g) in WT and *hs1* plants measured in the morning and afternoon. Values represent mean ± SD from 3 biological replicates. **P < 0.01, *P < 0.05; ns, not significant (*P* > 0.05; 2-tailed Student's *t*-test).

### *HS1* encodes a myosin protein

To determine whether the causal mutation of the *hs1* is dominant or recessive, *hs1* was crossed with WT. All 43 F_1_ individuals exhibited a WT phenotype without any leaf damage in the field conditions, indicating that the causal mutation is recessive and that the WT allele complements the mutant phenotype in heterozygous F_1_ plants (Fig. S11a). In the subsequent F_2_ population, leaf scorching and WT phenotypes segregated at a ratio of 20:73, consistent with the expected 1:3 Mendelian ratio (*χ*^2^ = 0.61, *P* = 0.44). Together, these segregation patterns demonstrated that the leaf scorching phenotype in *hs1* is governed by a single recessive mutation.

Next, BSA of the F_2_ population was performed with 4 biological replicates over 2 years to identify the causal mutation (Fig. S11b). Two genes (*Sobic.002G340200* and *Sobic.002G339300*) on chromosome 2 were consistently identified in the 4 replicates. Screening alternative alleles of these 2 genes across our sequenced 1,256 mutants (Jiao et al. 2024) revealed that only *Sobic.002G339300* carried 4 independent alleles producing a similar leaf scorching phenotype (Fig. 4a; Fig. S11c). To further validate *Sobic.002G339300* as the causal gene, a complementation *(*cis*–*trans*)* test was conducted by crossing homozygous *hs1* with the mutant *25M2-1883*. All F_1_ offspring displayed the leaf scorching phenotype (Fig. 4b), demonstrating noncomplementation between the 2 mutants. Thus, replicated BSA mapping, independent allelic evidence, and complementation testing together confirmed *Sobic.002G339300* as the causal gene, designated *HS1.*

**Figure 4:**
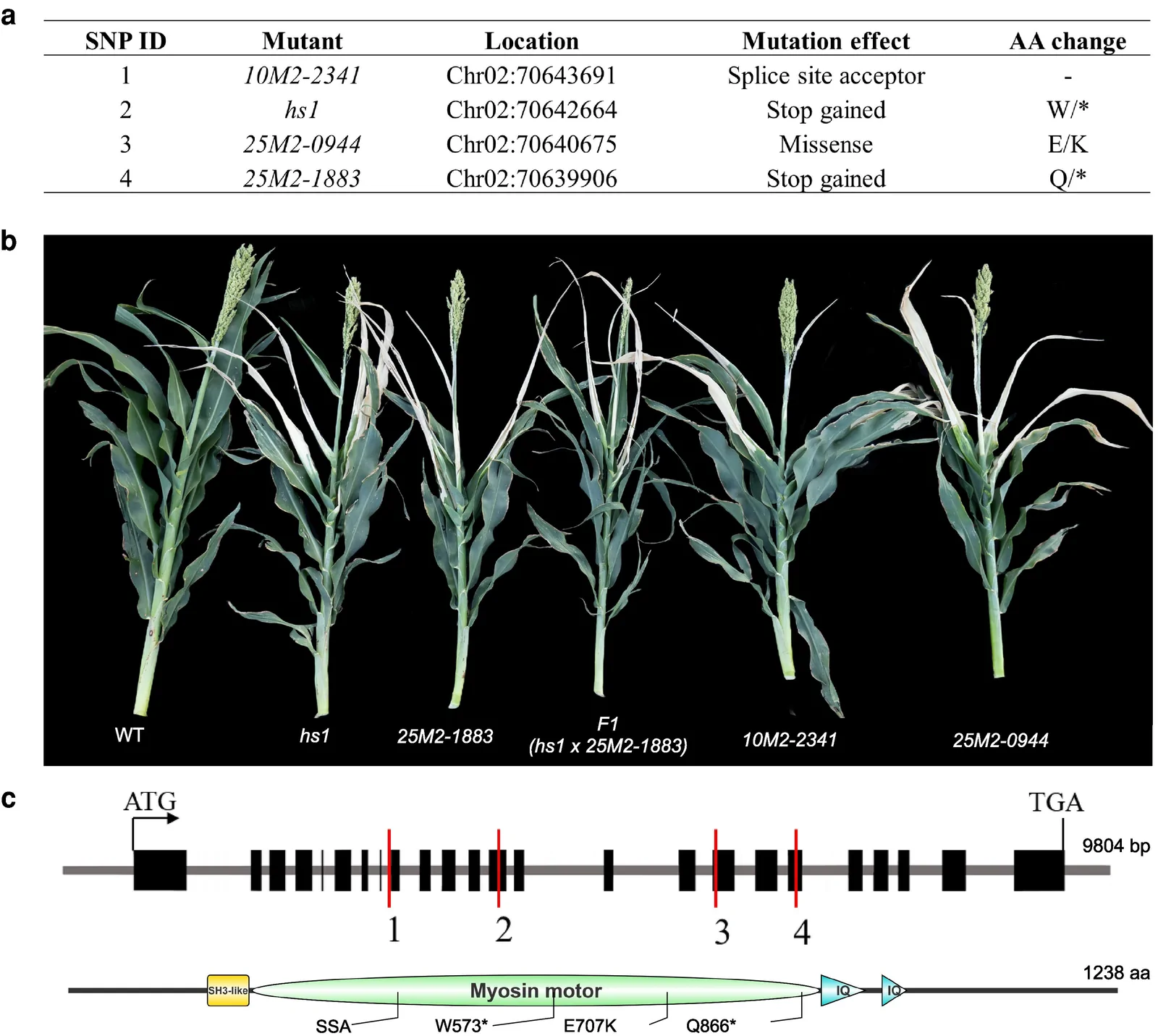
Identification of the causal gene for the *hs1* mutant. a) Four independent alleles of the candidate gene *Sobic.002G339300.* b) Complementation test to validate the causal gene. Representative phenotypes of WT, *hs1*, *25M2-1883*, the F1 progeny from the *hs1* × *25M2-1883* cross, and 2 additional independent mutant alleles (*10M2-2341* and *25M2-0944*) showing leaf scorching. c) Gene structure and protein domains of *Sobic.002G339300* (myosin protein). Positions of the 4 independent mutations shown in (a) are indicated. SSA indicates a splice-site acceptor mutation, and * indicates a stop codon.

The WT *HS1* gene encodes a myosin protein and consists of 3 major functional domains: src Homology-3 (SH3)-like, motor domain, and isoleucine–glutamine (IQ) motif-containing neck region (Fig. 4c). Structural modeling of the *hs1* mutant alleles revealed substantial disruption of protein architecture, including truncation of the tail region and pronounced disorder within the head and neck regions (Fig. S12a to c). The *hs1* allele introduced a premature stop codon at the 573rd amino acid, and structural alignment with the WT protein revealed significant conformational deviation (TM-score = 0.34/0.73; RMSD = 1.22 Å). These variants in protein structure strongly suggest impaired *HS1* motor function in the mutant background.

### *HS1* encodes a myosin VIII with a grass-specific structural feature

Plant myosins are classified into 2 classes: myosin XI and VIII. The phylogenetic analyses of these classes across *Sorghum bicolor* (12 genes), *Z. mays* (8 genes), *O. sativa* (8 genes), and *A. thaliana* (17 genes) classified *HS1* as a member of the myosin VIII class (Fig. S13). We next expanded our phylogenetic survey across plants. No intact myosin VIII class proteins were detected in surveyed algal lineages (Dataset S1), consistent with previous reports (Richards and Cavalier-Smith 2005; Peremyslov et al. 2011; Mühlhausen and Kollmar 2013; Sebe-Pedros et al. 2014; Nebenführ and Dixit 2018). In contrast, myosin VIII was identified throughout land plants (Dataset S1). We compiled 134 myosin VIII class proteins from 44 species spanning early-diverging land plants to angiosperms and constructed a phylogenetic tree using 3 *Arabidopsis* myosin XI class proteins as an outgroup.

The phylogenetic tree showed that myosin XI proteins branched outside the myosin VIII clade, defining the root of the myosin VIII lineage (Fig. 5a; Fig. S14). Within the myosin VIII class, lycophyte sequences occupied basal positions, followed by bryophytes, which formed a sister clade to angiosperms (Fig. 5a). Angiosperm myosin VIII proteins segregated into 2 major paralogous clades, designated VIII-A and VIII-B, reflecting an ancient duplication near the base of angiosperms (Peremyslov et al. 2011; Mühlhausen and Kollmar 2013). Within each clade, basal angiosperm sequences preceded a clear divergence between monocot and eudicot lineages (Fig. 5a). Within each monocot lineage, grass sequences formed distinct subclades. *HS1* clustered within the grass-specific VIII-B clade, which contained 16 members from 11 grass species (Fig. 5a; Fig. S15), compared with 17 members from 12 species in the grass VIII-A clade. These results indicate that *HS1* is a grass-specific myosin VIII protein.

**Figure 5:**
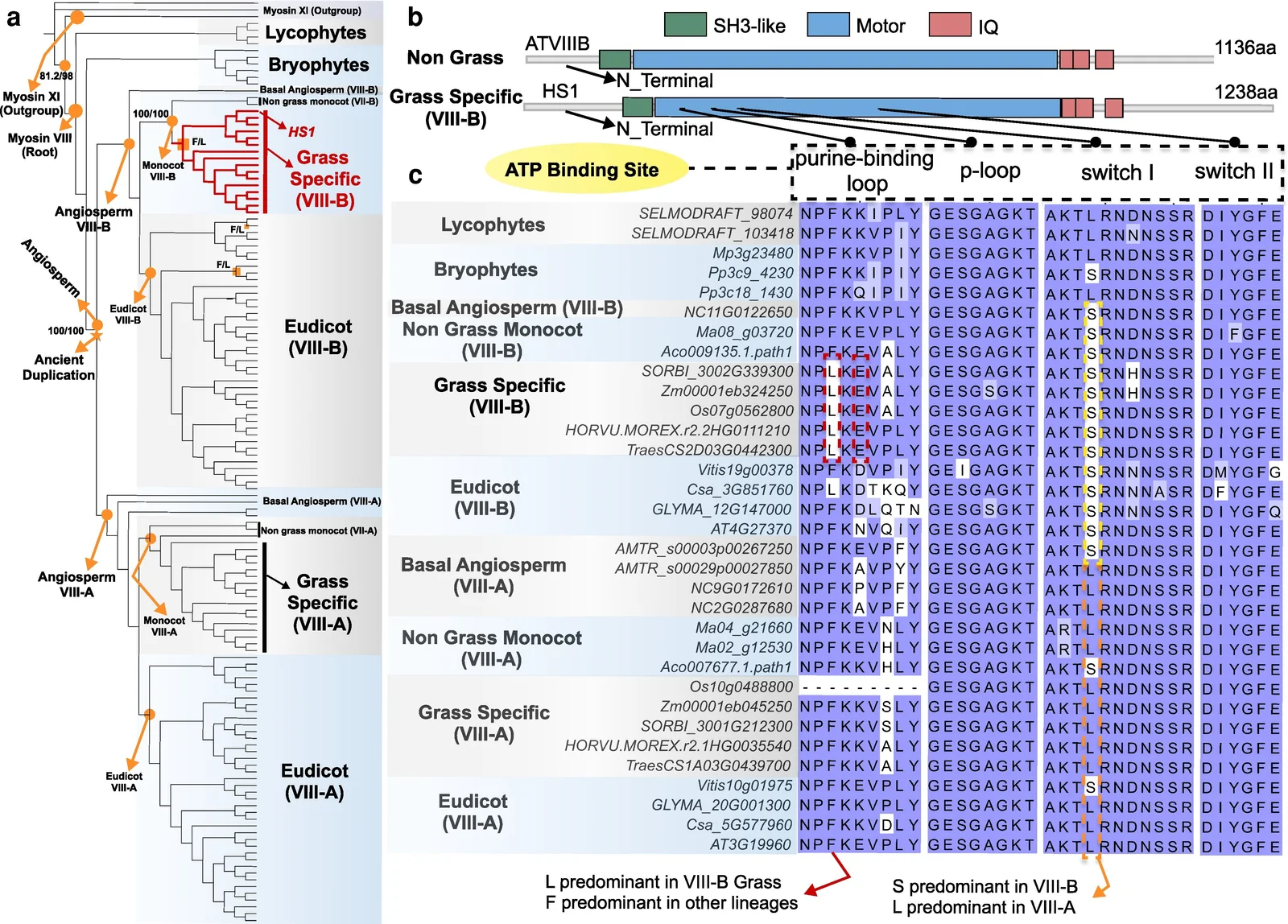
Phylogenetic analysis of myosin proteins in plants. a) The phylogenetic tree of the myosin family in plants. Branch support values are shown as SH-aLRT test/bootstrap; the circle indicates the lineage of the node. The square indicates the F/L substitutions in class myosin VIII. b) Schematic representation of the protein domain architecture of nongrass myosin VIII (*A. thaliana* myosin VIII-B [ATVIIIB] as representative) and grass-specific myosin VIII-B (HS1 as representative). c) Multiple sequence alignment of the ATP-binding region of class VIII myosins from representative plant lineages. Lineage groupings are shown on the left. ATP-binding sites (purine-binding loop, *P*-loop, switch I, and switch II motifs) are indicated above the alignment. Conserved residues are shaded in the alignment. Dashed boxes highlight lineage-specific sequence features.

Accordingly, angiosperm myosin VIII proteins were grouped into 4 major clades (each represented by >10 species) for subsequent analyses: grass-specific VIII-A, grass-specific VIII-B, eudicot VIII-A, and eudicot VIII-B (Fig. 5a). Among these clades, the grass-specific VIII-B clade exhibited the highest IUPred2 scores at both the N- and C-terminal regions (peak_N and peak_C = 0.81) (Fig. S15a; Dataset S2) and the longest median N-terminal intrinsically disordered region (IDR; 130.5 aa) (Fig. S15a and b; Dataset S3), together indicating a strong propensity for intrinsic disorder (Mészáros et al. 2018). Notably, the grass-specific VIII-B clade also displayed the longest median N-terminal length (181.5 aa) and uniquely retained both Motif 1 and Motif 2 (present in >90% of clade members), whereas other clades conserved only a single motif (Fig. 5b; Fig. S16). Collectively, these features suggest that grass-specific VIII-B myosins possess extended and disordered N-terminal regions that may facilitate dynamic protein–protein interactions or regulatory functions.

In addition to terminal disorder, grass-specific VIII-B myosins exhibited lineage-specific substitutions within the motor domain (Fig. 5b and c). Within the conserved purine-binding loop (NPxxxxxY), a leucine (L) at the third position was consistently present in grass-specific VIII-B myosins, as well as in cucurbit species and *Kalanchoe fedtschenkoi*, whereas all other plant lineages retained phenylalanine (F) (Fig. 5; Dataset S4). This L residue was consistently paired with a downstream glutamate (E), forming an L–E combination exclusive to grass-specific VIII-B myosins (Fig. 5c; Dataset S4). In addition, a leucine residue was uniquely conserved within the relay loop across grass-specific VIII-B (Fig. S17). These features were absent from nongrass myosin VIII proteins.

Structural modeling further revealed that *HS1* exhibited an estimated free energy of ATP binding of 2.267 kcal/mol, compared with 18.338 kcal/mol for *Arabidopsis* myosin VIII-A ATM1 (*AT3G19960*) and 6.677 kcal/mol for soybean myosin VIII-B (*GLYMA_12G147000*), indicating more favorable ATP binding in *HS1*, with approximately 8.1-fold and 3.0-fold lower ΔG values relative to ATM1 and soybean VIII-B, respectively (Fig. S18). The F to L substitution occurs within the conserved purine-binding loop, a core component of the ATP-binding pocket (Risal et al. 2004), whereas other ATP-binding residues remained conserved across myosin VIII groups (Fig. 5c). These lineage-specific substitutions likely influence nucleotide binding, suggesting selective modifications of motor mechanics or grass-specific regulation.

Together, these lineage-specific features, including grass-specific N-terminal intrinsic disorder and motor-domain substitutions, indicate a distinct evolutionary trajectory for *HS1* that coincides with the divergence of Poaceae from other monocots.

### SCW-associated genes upregulated in *hs1* developing leaf

Analysis of publicly available transcriptome datasets (Goodstein et al. 2012; Papatheodorou et al. 2018) revealed that *HS1* was highly expressed (Fragments Per Kilobase of transcript per Million mapped reads, FPKM > 30) in sorghum leaves, inflorescences, and roots across multiple vegetative and reproductive stages, suggesting a broad role for *HS1* in sorghum development (Fig. S19). To investigate transcriptome changes specifically in the developing leaf of the *hs1* mutant, we performed RNA-seq on the *hs1* line, which carries a nonsense mutation introducing a stop codon at amino acid position 573. Leaf base samples of developing and mature leaves with 3 biological replicates were collected at the sorghum GPD stage in the morning under optimal temperature and irrigation (Fig. 6a).

**Figure 6:**
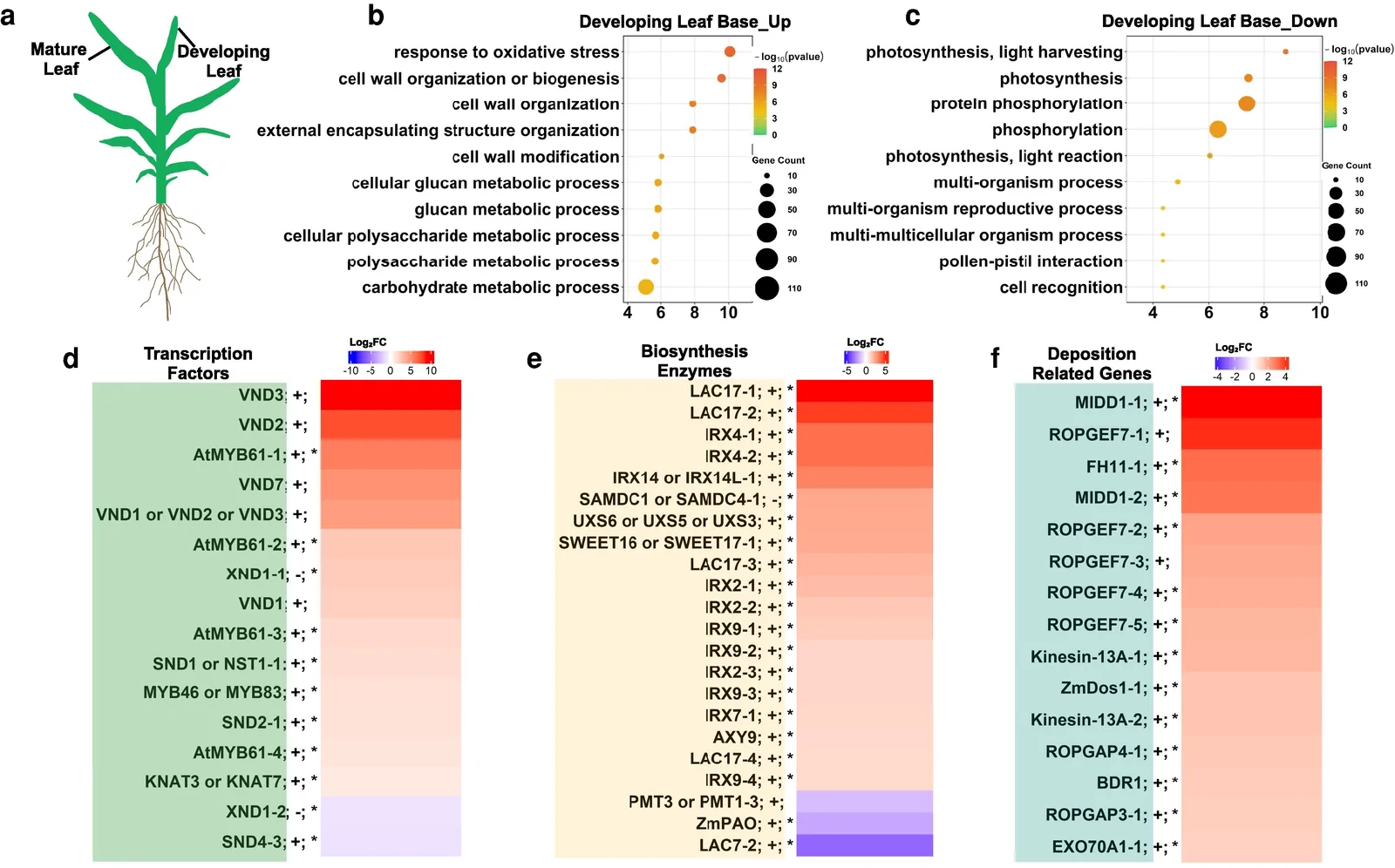
Transcriptome analysis of the midrib at the base of developing leaves in WT and *hs1*. a) Schematic diagram showing the leaf whose basal midrib was used for transcriptome analysis. b, c) GO enrichment analysis of upregulated (b) and downregulated (c) genes in the developing leaf base midrib of *hs1* compared with WT. The top 10 GO terms (*P* < 0.01), ranked by *P*-value. d−f) Heatmaps showing log 2-fold changes in sorghum ortholog expression in *hs1* relative to WT for known SCW-related genes from *A. thaliana*, *Z. mays*, and *O. sativa*, grouped as transcription factors d), biosynthesis enzymes e), and deposition-related genes f); genes with |log 2-fold change| > 1 are shown. “+” and “–” indicate positive and negative regulators of SCW formation, respectively. *q-value <0.05. FC, fold change. The complete dataset is provided in Fig. S20 and Supplementary Dataset S5.

Compared with WT, a total of 2,383 upregulated and 1,242 downregulated differentially expressed genes (DEGs) (|log_2_FC| > 1) were identified in developing leaves of *hs1*, whereas only 729 upregulated and 522 downregulated DEGs (|log_2_FC| > 1) were detected in mature leaves (Fig. S20a and b). Consistent with the developmental onset of the *hs1* phenotype, more DEGs were detected in developing leaves than in mature leaves. Top 10 upregulated GO terms in the developing leaf base were enriched in cell wall–associated processes, such as cell wall biogenesis, organization, and modification (Fig. 6b). Five out of top 10 downregulated GO terms were enriched in photosynthesis processes (Fig. 6c). In contrast, upregulated genes in the mature leaf were enriched in general metabolic processes, such as RNA biosynthesis and macromolecule biosynthesis (Fig. S20c). In addition, downregulated DEGs were associated with transmembrane transport in mature leaves (Fig. S20d).

To investigate whether *HS1* interfaces with known regulatory networks controlling PX SCW formation, we analyzed transcriptional profiles of 128 sorghum homologs of characterized xylem SCW pathway genes from *A. thaliana*, *Z. mays*, and *O. sativa*. This set included 38 transcription factors (TFs), 53 biosynthetic enzymes (EZs), and 37 deposition-related (DP) genes (Dataset S5). Strikingly, 99 (77.34%) genes were upregulated in *hs1*, with 64 exhibiting statistically significant induction [false discovery rate (FDR) *P* < 0.05] (Fig. 6d to f; Fig. S21; Dataset S5).

The coordinated upregulation of SCW-associated genes in *hs1* background indicates a transcriptional reprogramming due to defective vascular structure. Despite robust activation of upstream regulators and biosynthetic enzymes, PX vessels in *hs1* failed to maintain structural integrity. We therefore hypothesized that *HS1* is not required for the transcriptional activation of SCW genes but is essential for the effective execution of wall assembly. These results position *HS1* downstream of the core transcriptional network, where it likely facilitates cytoskeletal or mechanical processes that translate SCW gene expression into functional PX reinforcement during leaf development.

### *HS1* regulates secondary wall integrity through lignin-associated pathways

Myosins function as molecular motors that transport diverse cellular cargos, including components associated with cell wall biosynthesis (Zhang et al. 2019). To determine which cell wall–related processes are associated with *HS1*, we reanalyzed a published sorghum single-cell RNA-seq (scRNA-seq) dataset of leaf tissues (Swift et al. 2024). In this dataset, during first leaf development (0 to 48 h after germination, HAG), *HS1* showed the highest tissue expression specificity in the xylem cells compared with other cell types (Fig. 7a). In contrast, the sorghum myosin VIIIA homolog Sobic.001G212300 showed the strongest expression in epidermal cells and only low expression in xylem cells (Fig. S22). Within the xylem cells, the highest expression of HS1 was detected at 12HAG, when the first leaf is rapidly developing, compared with other timepoints. However, its expression decreased when the first leaf had fully matured (48HAG) (Fig. 7b; Dataset S6). In another single-cell dataset, *HS1* expression remained low in mature leaf xylem (D’Agostino et al. 2025). This temporal and cell-type–specific expression pattern places *HS1* at the onset of PX differentiation in developing leaves. Consistent with this pattern, the 3 most highly expressed xylem SCW biosynthetic genes at 12 HAG were all associated with lignification, including *irregular xylem 4* (IRX4/CCR1; Sobic.002G146000), *laccase 7* (LAC7; Sobic.003G357500), and *irregular xylem 9* (IRX9; Sobic.009G026101). Expression of these genes declined as leaves matured (48 HAG) (Fig. 7c; Dataset S7). Notably, of these 3 genes, *irregular xylem 4* (IRX4/CCR1; Sobic.002G146000) and *laccase 7* (LAC7; Sobic.003G357500) belong to the lignin-specific biosynthetic pathway (Vanholme et al. 2010). In *Arabidopsis*, *CCR1* encodes a cytosolic cinnamoyl-CoA reductase (CCR) functioning in the cytosol, whereas *LAC7* encodes a laccase that catalyzes monolignol polymerization in SCW; both are required for the lignification of xylem SCW (Laskar et al. 2006; Zhao et al. 2015). In our bulk RNA-seq data, *CCR1 (IRX4)* was significantly upregulated (log_2_FC = 3.75), whereas *LAC7* showed a strong downregulation in *hs1* (log_2_FC = −3.44) (Dataset S4). This contrast may reflect compensatory upregulation of cytosolic lignin biosynthesis while apoplastic lignin polymerization is impaired in *hs1*. We also examined the broader set of lignin-specific SCW genes detected in the single-cell dataset. Of the 10 lignin-specific genes, 9 showed higher expression in myosin cells than in normal cells at 12 HAG (Fig. 7c; Dataset S7). These suggested that *HS1* was likely involved in lignin synthesis within the xylem of developing leaves.

**Figure 7:**
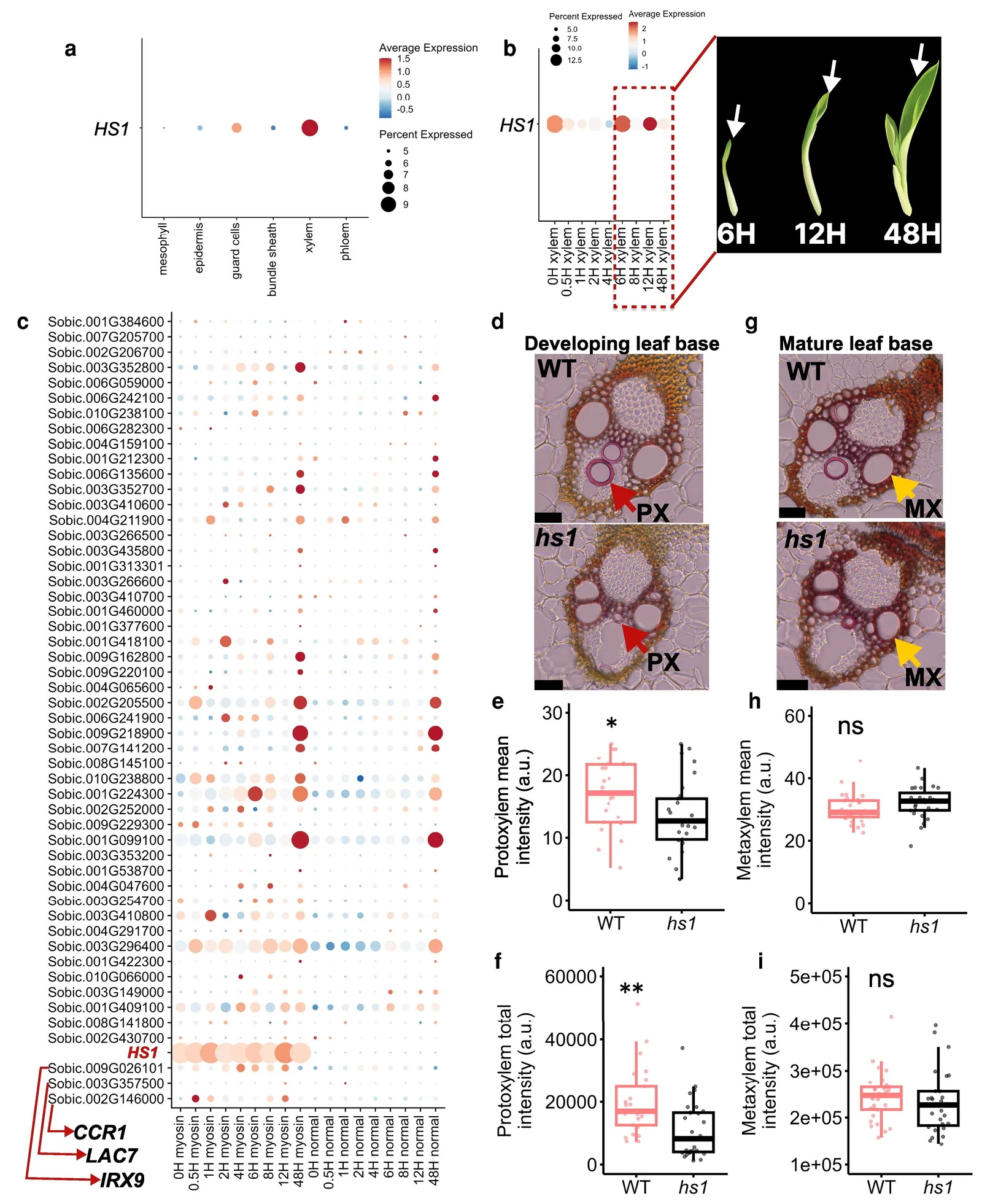
Coordinated single-cell expression of *HS1* and SCW biosynthetic genes during leaf development and corresponding alterations in xylem lignification in *hs1* leaf midribs relative to WT. a) Dot plot of single-cell *HS1* expression across sorghum leaf cell types during first leaf development (0 to 48 h after germination, HAG). b) Dot plot showing single-cell *HS1* expression in xylem cells across sorghum leaf developmental time points. The schematic at right depicts first-leaf morphology at 6 h (growth initiation), 12 h (rapid growth), and 48 h (maturation) (modified from Swift et al. 2024), with arrows indicating the first leaf. H, hours after germination. c) Dot plot of coordinated single-cell expression patterns of *HS1* and SCW biosynthetic genes in xylem cells across sorghum leaf developmental time points. Genes are ordered on the *y*-axis by average expression at 12 HAG, with the highest expression positioned at the bottom. *X*-axis labels indicate time points from 0 to 48 HAG. “Myosin” denotes xylem cells exhibiting high *HS1* expression, whereas “normal” denotes xylem cells exhibiting low *HS1* expression at the corresponding time points. Genes with high expression and patterns similar to *HS1* are indicated by arrows. d−i) Phloroglucinol-HCl staining showing lignin intensity in developing and mature leaf base midribs of WT and *hs1*. Developing leaf bases: cross-sections (d), PX lignin mean intensity (e), and total intensity (f). Mature leaf bases: cross-sections (g), MX lignin mean intensity (h), and total intensity (i). Arrows in panels (d) and (g) indicate PX and MX, respectively. Boxes indicate the first and third quartiles, and horizontal lines indicate the median. **P < 0.01; *P < 0.05; ns, not significant (*P* > 0.05); 2-tailed Student's *t*-test. Bars = 50 µm. Data were collected from 3 biological replicates for each genotype. Quantification was performed on individual PX or MX vessels pooled across biological replicates (*n* ≥ 25 for d and e; *n* ≥ 27 for g and h).

To validate, we compared the lignin intensity in the xylem of developing leaves and mature leaves in WT and *hs1*. Lignin staining revealed visibly weaker signals in *hs1* PX of developing leaves (Fig. 7d). Quantitative analysis revealed a significant reduction in *hs1* compared with WT, with a 21% decrease in mean lignin intensity and a 45% decrease in total lignin signal (*P* < 0.05 and *P* < 0.01, respectively; Fig. 7e and f). In contrast, no significant difference (*P* > 0.05) was detected in mature leaves MX SCW (Fig. 7g to i). Together, these results demonstrate that *HS1* is specifically required for proper lignin deposition during PX SCW formation in developing leaves. The developmental specificity of the defect, combined with transcriptional misregulation of lignin polymerization genes, supports a role for HS1 in proper lignin deposition, likely by facilitating the trafficking of SCW components, thereby contributing to PX integrity and water transport.

### Low genetic diversity of *HS1* in the sorghum population

Given the essential role of HS1 in SCW development and PX integrity, we next examined nucleotide diversity (*π*) at the *HS1* locus and its 20 kb flanking regions in the sorghum population. Nucleotide diversity was calculated using 400 Sorghum Association Panel (SAP) accessions (Bred, Conv, and Other) together with 46 wild sorghum from the SorGSD database (Dataset S8) (Morris et al. 2013; Liu et al. 2021; Boatwright et al. 2022). Notably, *π* was high across most of the flanking regions in all accessions but declined sharply in the *HS1* locus in both cultivated and wild sorghum (Fig. 8a). Across SAP subgroups, diversity at *HS1* was reduced by approximately 7-fold compared to the immediately adjacent 20 kb regions, a pattern consistent with purifying selection or reduced neutral diversity at this locus. This pattern indicates that HS1 has experienced intense selective pressure in the sorghum population, likely preserving its function in maintaining vascular robustness under the heat- and drought-prone conditions typical of sorghum-growing environments.

**Figure 8:**
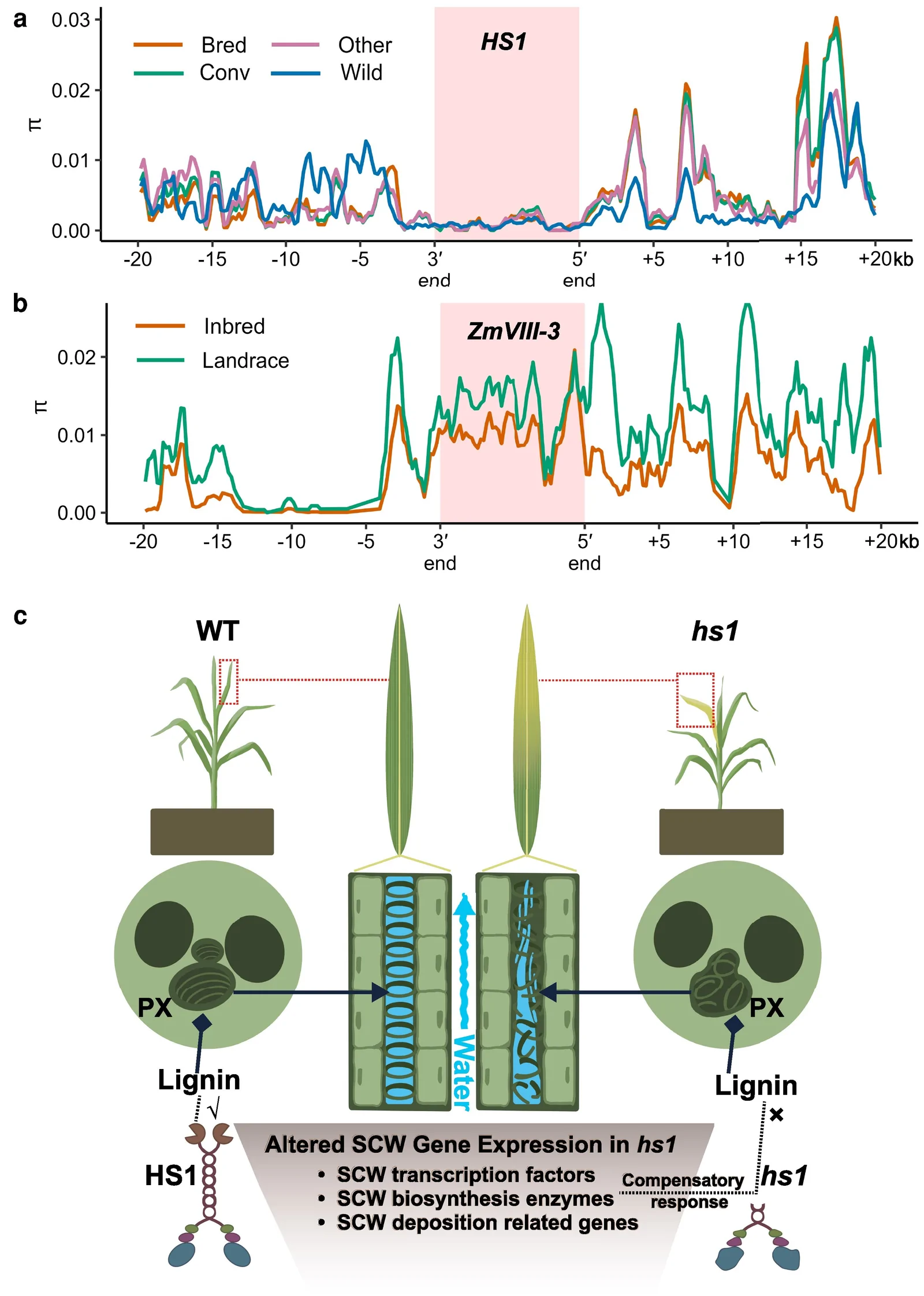
Evolutionary conservation and functional model of *HS1* in maintaining PX secondary cell wall (SCW) integrity. a) Nucleotide diversity (*π*) across the *HS1* locus and its 20 kb flanking regions in breeding lines (Bred), photoperiod-converted lines (Conv), wild accessions (Wild), and accessions without subgroup assignment (Other). The shaded region denotes the HS1 gene body. b) Nucleotide diversity (*π*) across *ZmVIII-3* and its 20 kb flanking regions. The shaded region denotes the *ZmVIII-3* gene body. c) Proposed model for *HS1* function in maintaining PX secondary cell wall (SCW) integrity. The altered SCW gene expression in *hs1* may reflect a compensatory response to impaired wall formation. Loss of *HS1* leads to reduced lignin, PX collapse, and impaired water transport. PX indicates PX vessels.

In striking contrast, the maize homolog at the *ZmVIII-3* (Zm00001d021785) locus exhibited the opposite pattern, with consistently high nucleotide diversity maintained across diverse maize populations (Fig. 8b). This suggests differential evolutionary trajectories for this conserved gene between the 2 species, possibly reflecting differences in selective pressures related to vascular development or environmental adaptation. Given the adaptation of sorghum to hot and drought-prone environments, where hydraulic efficiency is critical for plant performance, the reduced diversity at the HS1 locus is consistent with stronger evolutionary constraint. By contrast, the higher diversity observed in maize, a species generally cultivated under comparatively more mesic conditions, may reflect differences in environmental selection pressures acting on vascular function, although demographic history may also contribute. Together, the strong evolutionary constraint at the *HS1* locus further supports a central role of *HS1* in maintaining vascular robustness and plant hydraulic function (Fig. 8c).

## Discussion

### HS1 is essential for PX integrity and leaf hydraulic function

In this study, we demonstrated that the sorghum heat-sensitive phenotype of the *hs1* mutant was caused by the impaired water transport. Leaf scorching was observed in the field and also in controlled environments when the transpiration increases, such as at noon in the greenhouse. This phenotype was similar to maize drought-overly-sensitive1 (*ZmDos1*) and drought-sensitive1 (*ds1*) mutants (Huang et al. 2024; Zhu et al. 2026). Further investigation revealed that root and stomatal traits were unchanged in *hs1* compared with WT (Figs. S3 and S4), similar to the pattern in *Zmdos1* (Huang et al. 2024). In contrast, dye uptake and movement assays, which have been used in previous studies to localize organ-level water transport defects (Dong et al. 2020; Li et al. 2023; Huang et al. 2024), revealed restricted longitudinal safranin movement in developing *hs1* leaves (Fig. 2; Fig. S5), suggesting impaired internal water transport.

As xylem is the primary pathway for water transport, we examined its structure. Samples were collected before visible leaf scorching and at night to minimize transpiration-induced xylem tension (Chen et al. 2005). Collapsed PX elements were still observed, and serial sections confirmed that this defect was consistently present at the base of developing leaves (Fig. S23), indicating a preexisting developmental PX defect rather than stress-induced damage. As transpiration increased at noon or under heat treatment, these preexisting defects became physiologically evident as leaf scorching (Fig. 3; Fig. S10). Detached leaf of *hs1* also showed slower water loss than WT (Fig.S9d to f), similar to the observation in *lew2-1* (Chen et al. 2005). These physiological and microscopic evidence suggest that HS1 is required for PX structure integrity and function.

RNA-seq samples collected in the early morning, before visible scorching and under low transpiration, showed that most of the top enriched GO terms were related to cell wall-associated processes rather than stress responses (Fig. 6b). This is consistent with previous reports that myosin VIII proteins are involved in cell wall–related processes, plasmodesmatal activity, and cell plate maturation, suggesting a broader role in coordinating membrane and wall dynamics (Reichelt et al. 1999). Single-cell transcriptomic analyses further revealed xylem-enriched expression of HS1 and its association with lignin-specific gene expression (Fig. 7c). Given that lignin deposition is fundamental to xylem mechanical strength and water transport, and that altered lignification can compromise hydraulic conductance and vessel integrity (Boyce et al. 2004; Voelker et al. 2011), these raise the possibility that HS1 may contribute to lignin-associated xylem maturation, thereby influencing xylem function and water transport. Together, these findings support a model in which the *hs1* phenotype originates from PX defects and impaired hydraulic function, with increased transpirational demand acting as a trigger for leaf scorching.

### Potential impact of HS1 discovery

Efficient water transport is essential for plant growth and environmental resilience, yet direct evidence linking motor proteins to this process has remained limited. Here, we identify HS1, a grass-specific myosin VIII protein, as an essential regulator of PX integrity and hydraulic function in sorghum. Previous studies of plant motor proteins have focused mainly on model plants and cellular or subcellular processes (Reddy and Day 2001a, 2001b; Hussey et al. 2002; Smith 2003; Sparkes 2011; Nebenführ and Dixit 2018). Recent work showed that formin-mediated actin polymerization is required for SCW banding in PX (Kijima et al. 2025). Our findings extend this framework by linking an actin-based motor to PX SCW integrity and water transport. Because grasses produce long, rapidly elongating leaves with high transpirational demand, sorghum provides a valuable system for uncovering hydraulic regulators not readily predicted from Arabidopsis-based paradigms (Karam 2005; Caldeira et al. 2014; Scoffoni et al. 2017). Notably, reduced nucleotide diversity at the HS1 locus across sorghum populations (Fig. 8a) further suggests strong evolutionary constraint, consistent with a role for HS1 in sorghum hydraulic adaptation.

HS1 belongs to a grass-specific myosin VIII-B lineage characterized by an extended intrinsically disordered N-terminal region and conserved VIII-B motifs (Figs. S16 and S17), which may facilitate protein–protein interactions during xylem development. Structural modeling predicted stronger ATP-binding affinity in HS1 and ZmVIII-3 than in their Arabidopsis and soybean homologs (Fig. S20), suggesting a potentially grass-specific conserved modification of motor activity. Together, these findings suggest that lineage-specific structural features and altered motor properties may have supported functional specialization during vascular evolution.

Finally, the sustained developmental impact of HS1 underscores its central role in maintaining xylem performance and highlights its agricultural relevance. Loss of HS1 disrupts PX integrity, causing hydraulic and physiological defects across developmental stages and multiple leaves (Fig. S7). These findings underscore the central role of HS1 in maintaining xylem integrity and hydraulic function and position it as a promising target for crop improvement. Leveraging HS1 function or natural allelic variation may offer new routes to enhance water transport and stress tolerance in grass crops under thermally and hydrologically challenging environments. Thus, HS1 links fundamental xylem developmental mechanisms with translational opportunities for crop resilience.

### Outstanding questions and future directions

While *HS1* established a functional framework for actin-motor protein activity in the xylem, several mechanistic questions remain. Foremost is the mechanism by which HS1 maintains PX integrity remains unresolved. Based on our transcriptome data and previous studies of plant myosins, we propose that HS1 may affect PX SCW formation through trafficking or positioning of SCW-associated machinery, rather than acting primarily as a direct transcriptional regulator. In *hs1*, many genes encoding cytosolic enzymes involved in SCW or lignin-related metabolism were upregulated, which may reflect a compensatory response to impaired wall formation. In contrast, several SCW-associated genes acting proximal to the plasma membrane or cell wall were downregulated, consistent with likely defective local organization or delivery of wall-related components (Dataset S9). This possibility is consistent with previous reports that class VIII myosins localize to the plasma membrane (Symonds et al. 2024). In addition, motor proteins are key drivers of intracellular cargo trafficking along cytoskeletal tracks (Cai and Cresti 2012). Therefore, HS1 may contribute to trafficking instead of transcription. Future live-cell imaging of HS1–cargo interactions and immunolocalization of SCW enzymes in *hs1* will help distinguish between cargo transport and structural scaffolding.

Additional questions concern the mechanistic basis of lineage-specific adaptations. *HS1* belongs to the myosin VIII family, which remains comparatively understudied relative to myosin XI (Ryan and Nebenführ 2018; Olatunji et al. 2023). Grass-specific VIII-B proteins are distinguished by extended N-terminal intrinsically disordered regions and conserved lineage-specific motifs. How these structural features modulate motor activity and contribute to PX SCW formation, hydraulic function, and stress adaptation warrants further investigation.

Finally, the discovery of HS1 raises broader questions about motor-based regulation of vascular development in grasses. In sorghum, HS1 defines a protein with grass-specific feature required for PX integrity and hydraulic function, suggesting that related proteins in maize, rice, and other grasses may have unexplored roles in xylem development and water transport. Moreover, HS1 appears functionally distinct from Arabidopsis ATM1 (Olatunji et al. 2023), and this contrast suggests that different myosin VIII lineages may have acquired branch-specific functions adapted to distinct developmental or physiological contexts. Determining whether other myosin VIII branches (Fig. 5a) show similarly specialized roles will provide broader insight into the evolution of motor protein function and its potential relevance for crop improvement.

## Supplementary Material

kiag712_Supplementary_Data

## Data Availability

All sequencing data have been deposited in the NCBI SRA database, including transcriptome data (BioProject PRJNA1419276) and whole-genome genomic DNA sequencing data of the mutants (SRR18888791, SRR18888802, SRR18888804, and SRR18888806).
