# Supplementary material for: A grass-specific structural myosin VIII regulates protoxylem development and hydraulic conductance in sorghum
Source: Plant Physiol. 2026 Sep 22;202(2):kiag712. doi: 10.1093/plphys/kiag712 (PMC13652027; doi:10.1093/plphys/kiag712)
Supplement: Supplementary file 1 [file kiag712_supplementary_data.zip › Supplemental_Methods.docx]

**Supplementary Method**

The following supplementary method is available for this article:

**Methods S1.** Bulked segregant analysis and mutation identification

**Methods S2.** Physiological and morphological analyses

**Methods S3.** RNA-seq data processing and analysis

**Methods S4.** Phylogenetic analysis

**Methods S5.** Intrinsic disorder analysis using IUPred2A

**Methods S6.** Phloroglucinol staining and lignin quantification

**Methods S7.** Nucleotide diversity analysis of *HS1* and *ZmVIII-3* loci

**Methods S1. Bulked segregant analysis and mutation identification**

Identification of the causal mutation was performed using a previously established BSA pipeline (Jiao *et al.*, 2018a; Jiao *et al.*, 2018b; Wang *et al.*, 2021). Briefly, genomic DNA was extracted from leaf tissue of 20 F₂ individuals exhibiting the mutant phenotype, using a CTAB-based protocol as described previously (Xin & Chen, 2012). DNA samples were quantified, pooled in equal proportions, and used to construct an Illumina paired-end (150 bp) library, yielding an average genome coverage of ~12–15×. Sequencing reads were processed through our established workflow (Jiao *et al.*, 2018b) and aligned to the Sorghum bicolor BTx623 V3 reference genome (McCormick *et al.*, 2018) with Bowtie2 (Langmead & Salzberg, 2012). Variant calling was done using BCFtools (Narasimhan *et al.*, 2016). To specifically capture EMS-induced mutations, SNPs were filtered to retain only GC→AT transitions, exclude background polymorphisms identified in resequencing data from our BTx623 germplasm, and satisfy read depth (5–100) and homozygosity criteria. Functional impacts of filtered variants were annotated using SnpEff (Cingolani *et al.*, 2012). Candidate causal mutations were defined as variants predicted to cause amino acid substitutions, premature stop codons, or splice-site alterations. To assess allelic recurrence, candidate variants were queried against an indexed sorghum mutant database (Jiao *et al.*, 2024).

**Methods S2. Physiological and morphological analyses**

Root morphology and xylem sap exudation

Whole root systems were gently removed from the pots. Adhering soil was washed away under running tap water with great care to avoid mechanical damage and to preserve fine lateral roots. Cleaned roots were spread on black cardboard and photographed. The resulting images were analyzed by ImageJ (Schneider *et al.*, 2012) for root morphology and root surface area. For sap exudation measurements, plants at the same stage were cut 2 cm above the stem base. To minimize disturbance and water loss, the cut stem base was immediately covered with an inverted 50-mL centrifuge tube to maintain near-saturated humidity. Xylem sap was allowed to exude naturally without physical contact or applied pressure. Sap exudation was measured as the increase in weight of pre-weighed absorbent paper over 2 h.

Stomatal measurements

To obtain leaf epidermal impressions, a thin layer of clear nail polish was applied to the abaxial surface of developing leaves, allowed to dry, and lifted with transparent tape onto a glass slide. Stomatal images were captured using an EVOS M5000 microscope system. Stomatal density and stomatal size (represented as guard cell length) (Jordan *et al.*, 2015) were quantified in ImageJ (Schneider *et al.*, 2012). For each genotype, stomatal density was determined from three biological replicates, with at least three images analyzed per plant. Stomatal size was measured from 20 stomata per plant, with three biological replicates per genotype.

Water transport assays

For above-ground tissue assays, WT and *hs1* plants were cut at the base of the stem, and the entire above-ground portion was placed into 0.1% safranin solution on a sunny day. The total weight of the container, solution, and excised tissue was recorded at the start and again after 2 h to quantify dye uptake. For leaf assays, developing leaves were excised at the base and immersed in 0.1% safranin solution under the same conditions. The total weight was measured at the beginning and after 10 min to calculate dye absorption and movement. Three biological replicates were analyzed for each genotype.

Leaf water-loss assay

Developing leaves were detached from WT and *hs1* plants, with three biological replicates per genotype. Samples were placed at room temperature and weighed at 15, 30, and 45 min, and at 1 and 2 h. Water loss was calculated and expressed as the percentage of the initial fresh weight. Three biological replicates were analyzed for each genotype.

Leaf physiological parameters

Developing leaves from at least three biological replicates were used for each genotype. The H₂O₂, leaf relative water content (RWC), total chlorophyll (Chl), and electrolyte leakage (EL) assays were performed as previously described with minor modifications (Mostofa *et al.*, 2015; Mostofa *et al.*, 2020). To detect H₂O₂ accumulation in situ, samples were freshly cut and transferred to 0.1% 3,3-diaminobenzidine (DAB) for 72 h and then bleached in acetic acid–glycerol–ethanol (1/1/3) (v/v/v) solution for 24 h before photography. RWC was measured to assess leaf water status. Fresh leaves were cut and immediately weighed to obtain fresh weight (FW). Samples were fully hydrated by submerging them in deionized water for 24 h in 50-mL tubes placed on a horizontal shaker, after which turgid weight (TW) was recorded. Leaves were then dried at 70 °C for 72 h to obtain the dry weight (DW). RWC was calculated as:

RWC (%) = [(FW − DW) / (TW − DW)] × 100.

To determine chlorophyll content, 0.1 g leaves were homogenized in 2 mL chilled 80% acetone and centrifuged at 11,500 × g for 12 min at 4 °C. Absorbance of the clarified supernatant was measured at 663, 645, and 470 nm using 80% acetone as the blank. Total chlorophyll was calculated using a previously established formula (Arnon, 1949):

Total Chl content (mg g⁻¹ FW) = (20.2 × A645 + 8.02 × A663) × V / (1000 × W)

where V denotes the volume of 80% (v/v) acetone used for extraction (mL), and W represents the fresh weight of the tissue sample (g). EL was assessed by incubating ~0.3 g of 5-cm leaf segments with the midrib removed in 45 mL deionized water at room temperature on a horizontal shaker for 24 h. The conductivity of the bathing solution (C₁) was then measured, and the tubes were boiled for 30 min to release total electrolytes and obtain the final conductivity (C₂). EL was calculated as (C₁ / C₂) × 100.

Gas exchange measurements

WT and *hs1* plants’ stomatal conductance and E_apparent of the developing leaves (9th leaf) were measured with an LI-600 porometer (LI-COR, USA). Measurements were conducted in the morning (10 am) and again near noon (before the onset of leaf scorching symptoms). Five biological replicates were analyzed per genotype, with three measurements taken per replicate and averaged.

**Methods S3. RNA-seq data processing and analysis**

Clean RNA-seq reads were mapped to the Sorghum bicolor BTx623 reference genome (version 3) using STAR (Dobin *et al.*, 2013). Gene expression levels for each biological replicate were estimated as FPKM (fragments per kilobase of transcript per million mapped reads) using Cufflinks (Trapnell *et al.*, 2012). Reproducibility among biological replicates was evaluated by calculating Pearson correlation coefficients in R. To ensure data reliability, two replicates were excluded from downstream analyses because their coloration was below 0.95 when compared with other corresponding replicates. Differentially expressed genes (DEGs) were identified using the CuffDiff module of the Cufflinks package (Trapnell *et al.*, 2012). Functional enrichment analysis of DEGs was performed using agriGO Singular Enrichment Analysis with a hypergeometric test, applying a significance threshold of P < 0.01 (Tian *et al.*, 2017). Samples were collected with three biological replicates for each genotype.

**Methods S4. Phylogenetic analysis**

For the classification analysis (Supplementary Fig. S14), myosin VIII and XI sequences from Arabidopsis, maize, rice, and sorghum were included. Arabidopsis and maize sequences were obtained from published datasets (Reddy & Day, 2001; Wang *et al.*, 2014). Rice and sorghum sequences were retrieved from RAP-DB (Kawahara *et al.*, 2013; Sakai *et al.*, 2013) and Phytozome (Goodstein *et al.*, 2012), respectively. Sequences were aligned using ClustalW, and a neighbor-joining tree was constructed in MEGA 11.0.13 under the p-distance model with pairwise gap deletion and 1,000 bootstrap replicates (Tamura *et al.*, 2021; Madeira *et al.*, 2024). Trees were visualized in FigTree (Rambaut, 2014).

For the clade-level phylogenetic analysis of myosin VIII (Fig. 5A), 134 myosin VIII homologs from 44 land species were retrieved from Ensembl Plants via BioMart (Kinsella *et al.*, 2011; Yates *et al.*, 2022) by filtering the motor domain (MYSc_Myo8). Full-length sequences were aligned using the same method above. Maximum-likelihood inference was performed with IQ-TREE v3.0.1 using the ModelFinder-selected substitution model Q.PLANT+I+R6 under BIC (Kalyaanamoorthy *et al.*, 2017; Wong *et al.*, 2025). Branch support was evaluated with SH-aLRT and ultrafast bootstrap tests (1,000 replicates each) (Hoang *et al.*, 2018). Three Arabidopsis myosin XI proteins served as outgroups. Trees were visualized in iTOL (Letunic & Bork, 2024).

**Methods S5. Intrinsic disorder analysis using IUPred2A**

For each protein, residue-wise IUPred2 scores were calculated across the full-length sequence. To generate group-level disorder profiles, residue positions were normalized by protein length and binned into 100 equally sized relative-position bins along the protein. Mean IUPred2 scores were calculated for each bin within each group, producing averaged disorder profiles that reflect lineage-specific trends in intrinsic disorder distribution. Comparative analyses were restricted to groups containing more than ten species to ensure sufficient sampling depth.

**Methods S6. Phloroglucinol staining and lignin quantification**

At the GPD stage (~30 days after planting), fresh midrib tissues from the same basal region of mature and developing WT and *hs1* leaves were collected at night to reduce transpiration-driven xylem tension and embedded in Tissue Plus O.C.T. Compound (Fisher HealthCare, Cat# 4585, Houston, TX, USA). Samples were transversely sectioned at 30 µm thickness using a cryostat.

Fresh sections were transferred onto charged slides and rinsed with water for 2 min to remove residual O.C.T. The slides were then incubated in 2% phloroglucinol prepared in 95% ethanol for 5 min. Concentrated HCl was applied to induce the Wiesner reaction, and stained tissues were imaged immediately using a light microscope (Invitrogen EVOS M5000 Imaging System) under identical acquisition settings.

Lignin deposition was quantified in ImageJ (Schneider *et al.*, 2012) by measuring the mean and total staining intensity of protoxylem SCW in developing leaf-base midribs and of metaxylem SCW in mature leaf-base midribs, with all measurements corrected by background subtraction. Samples were collected with three biological replicates for each genotype.

**Methods S7. Nucleotide diversity analysis of HS1 and ZmVIII-3 loci**

Sorghum variants for 400 Sorghum Association Panel (SAP) accessions were obtained from the European Variant Archive (PRJEB51985) (Morris *et al.*, 2013; Boatwright *et al.*, 2022), and wild sorghum variants were retrieved from the Sorghum Genome SNP Database (SorGSD) (Liu *et al.*, 2021). Maize inbred and landrace variants were obtained from the unified high-coverage VCF dataset released by MaizeGDB (Andorf *et al.*, 2025) based on the B73 RefGen_v5 genome. Nucleotide diversity (π) was calculated using VCFtools v0.1.17 within 800-bp windows with a 200-bp step (Danecek *et al.*, 2011).

**Reference (Methods S1-S7)**

**Andorf CM, Ross-Ibarra J, Seetharam AS, Hufford MB, Woodhouse MR. 2025.** A unified VCF dataset from nearly 1,500 diverse maize accessions and resources to explore the genomic landscape of maize. *G3: Genes, Genomes, Genetics* **15**(2): jkae281.

**Arnon DI. 1949.** Copper enzymes in isolated chloroplasts. Polyphenoloxidase in Beta vulgaris. *Plant physiology* **24**(1): 1.

**Boatwright JL, Sapkota S, Jin H, Schnable JC, Brenton Z, Boyles R, Kresovich S. 2022.** Sorghum Association Panel whole‐genome sequencing establishes cornerstone resource for dissecting genomic diversity. *The Plant Journal* **111**(3): 888-904.

**Cingolani P, Platts A, Wang LL, Coon M, Nguyen T, Wang L, Land SJ, Lu X, Ruden DM. 2012.** A program for annotating and predicting the effects of single nucleotide polymorphisms, SnpEff: SNPs in the genome of Drosophila melanogaster strain w1118; iso-2; iso-3. *fly* **6**(2): 80-92.

**Danecek P, Auton A, Abecasis G, Albers CA, Banks E, DePristo MA, Handsaker RE, Lunter G, Marth GT, Sherry ST. 2011.** The variant call format and VCFtools. *Bioinformatics* **27**(15): 2156-2158.

**Dobin A, Davis CA, Schlesinger F, Drenkow J, Zaleski C, Jha S, Batut P, Chaisson M, Gingeras TR. 2013.** STAR: ultrafast universal RNA-seq aligner. *Bioinformatics* **29**(1): 15-21.

**Goodstein DM, Shu S, Howson R, Neupane R, Hayes RD, Fazo J, Mitros T, Dirks W, Hellsten U, Putnam N. 2012.** Phytozome: a comparative platform for green plant genomics. *Nucleic acids research* **40**(D1): D1178-D1186.

**Hoang D, Chernomor O, Von Haeseler A, Minh B, Le SV U. 2018.** Improving the ultrafast bootstrap approximation. *Molecular biology and evolution* **35**(2): 518-522.

**Jiao Y, Burow G, Gladman N, Acosta-Martinez V, Chen J, Burke J, Ware D, Xin Z. 2018a.** Efficient identification of causal mutations through sequencing of bulked F 2 from two allelic bloomless mutants of Sorghum bicolor. *Frontiers in plant science* **8**: 2267.

**Jiao Y, Lee YK, Gladman N, Chopra R, Christensen SA, Regulski M, Burow G, Hayes C, Burke J, Ware D. 2018b.** MSD1 regulates pedicellate spikelet fertility in sorghum through the jasmonic acid pathway. *Nature communications* **9**(1): 822.

**Jiao Y, Nigam D, Barry K, Daum C, Yoshinaga Y, Lipzen A, Khan A, Parasa SP, Wei S, Lu Z. 2024.** A large sequenced mutant library–valuable reverse genetic resource that covers 98% of sorghum genes. *The Plant Journal* **117**(5): 1543-1557.

**Jordan GJ, Carpenter RJ, Koutoulis A, Price A, Brodribb TJ. 2015.** Environmental adaptation in stomatal size independent of the effects of genome size. *New Phytologist* **205**(2): 608-617.

**Kalyaanamoorthy S, Minh BQ, Wong TK, Von Haeseler A, Jermiin LS. 2017.** ModelFinder: fast model selection for accurate phylogenetic estimates. *Nature methods* **14**(6): 587-589.

**Kawahara Y, de la Bastide M, Hamilton JP, Kanamori H, McCombie WR, Ouyang S, Schwartz DC, Tanaka T, Wu J, Zhou S. 2013.** Improvement of the Oryza sativa Nipponbare reference genome using next generation sequence and optical map data. *Rice* **6**(1): 4.

**Kinsella RJ, Kähäri A, Haider S, Zamora J, Proctor G, Spudich G, Almeida-King J, Staines D, Derwent P, Kerhornou A. 2011.** Ensembl BioMarts: a hub for data retrieval across taxonomic space. *Database* **2011**: bar030.

**Langmead B, Salzberg SL. 2012.** Fast gapped-read alignment with Bowtie 2. *Nature methods* **9**(4): 357-359.

**Letunic I, Bork P. 2024.** Interactive Tree of Life (iTOL) v6: recent updates to the phylogenetic tree display and annotation tool. *Nucleic acids research* **52**(W1): W78-W82.

**Liu Y, Wang Z, Wu X, Zhu J, Luo H, Tian D, Li C, Luo J, Zhao W, Hao H. 2021.** SorGSD: updating and expanding the sorghum genome science database with new contents and tools. *Biotechnology for Biofuels* **14**(1): 165.

**Madeira F, Madhusoodanan N, Lee J, Eusebi A, Niewielska A, Tivey AR, Lopez R, Butcher S. 2024.** The EMBL-EBI Job Dispatcher sequence analysis tools framework in 2024. *Nucleic acids research* **52**(W1): W521-W525.

**McCormick RF, Truong SK, Sreedasyam A, Jenkins J, Shu S, Sims D, Kennedy M, Amirebrahimi M, Weers BD, McKinley B. 2018.** The Sorghum bicolor reference genome: improved assembly, gene annotations, a transcriptome atlas, and signatures of genome organization. *The Plant Journal* **93**(2): 338-354.

**Morris GP, Ramu P, Deshpande SP, Hash CT, Shah T, Upadhyaya HD, Riera-Lizarazu O, Brown PJ, Acharya CB, Mitchell SE. 2013.** Population genomic and genome-wide association studies of agroclimatic traits in sorghum. *Proceedings of the National Academy of Sciences* **110**(2): 453-458.

**Mostofa MG, Fujita M, Tran L-SP. 2015.** Nitric oxide mediates hydrogen peroxide-and salicylic acid-induced salt tolerance in rice (Oryza sativa L.) seedlings. *Plant growth regulation* **77**(3): 265-277.

**Mostofa MG, Rahman MM, Siddiqui MN, Fujita M, Tran L-SP. 2020.** Salicylic acid antagonizes selenium phytotoxicity in rice: selenium homeostasis, oxidative stress metabolism and methylglyoxal detoxification. *Journal of hazardous materials* **394**: 122572.

**Narasimhan V, Danecek P, Scally A, Xue Y, Tyler-Smith C, Durbin R. 2016.** BCFtools/RoH: a hidden Markov model approach for detecting autozygosity from next-generation sequencing data. *Bioinformatics* **32**(11): 1749-1751.

**Papatheodorou I, Fonseca NA, Keays M, Tang YA, Barrera E, Bazant W, Burke M, Füllgrabe A, Fuentes AM-P, George N. 2018.** Expression Atlas: gene and protein expression across multiple studies and organisms. *Nucleic acids research* **46**(D1): D246-D251.

**Rambaut A 2014**. FigTree v1. 4.2, a graphical viewer of phylogenetic trees.

**Reddy AS, Day IS. 2001.** Analysis of the myosins encoded in the recently completed Arabidopsis thaliana genome sequence. *Genome biology* **2**: 1-19.

**Sakai H, Lee SS, Tanaka T, Numa H, Kim J, Kawahara Y, Wakimoto H, Yang C-c, Iwamoto M, Abe T. 2013.** Rice Annotation Project Database (RAP-DB): an integrative and interactive database for rice genomics. *Plant and Cell Physiology* **54**(2): e6-e6.

**Schneider CA, Rasband WS, Eliceiri KW. 2012.** NIH Image to ImageJ: 25 years of image analysis. *Nature methods* **9**(7): 671-675.

**Tamura K, Stecher G, Kumar S. 2021.** MEGA11: molecular evolutionary genetics analysis version 11. *Molecular biology and evolution* **38**(7): 3022-3027.

**Tian T, Liu Y, Yan H, You Q, Yi X, Du Z, Xu W, Su Z. 2017.** agriGO v2. 0: a GO analysis toolkit for the agricultural community, 2017 update. *Nucleic acids research* **45**(W1): W122-W129.

**Trapnell C, Roberts A, Goff L, Pertea G, Kim D, Kelley DR, Pimentel H, Salzberg SL, Rinn JL, Pachter L. 2012.** Differential gene and transcript expression analysis of RNA-seq experiments with TopHat and Cufflinks. *Nature protocols* **7**(3): 562-578.

**Wang G, Zhong M, Wang J, Zhang J, Tang Y, Wang G, Song R. 2014.** Genome-wide identification, splicing, and expression analysis of the myosin gene family in maize (Zea mays). *Journal of experimental botany* **65**(4): 923-938.

**Wang L, Lu Z, Regulski M, Jiao Y, Chen J, Ware D, Xin Z. 2021.** BSAseq: an interactive and integrated web-based workflow for identification of causal mutations in bulked F2 populations. *Bioinformatics* **37**(3): 382-387.

**Wong TK, Ly-Trong N, Ren H, Baños H, Roger AJ, Susko E, Bielow C, De Maio N, Goldman N, Hahn MW. 2025.** IQ-TREE 3: Phylogenomic Inference Software using Complex Evolutionary Models.

**Xin Z, Chen J. 2012.** A high throughput DNA extraction method with high yield and quality. *Plant methods* **8**: 1-7.

**Yates AD, Allen J, Amode RM, Azov AG, Barba M, Becerra A, Bhai J, Campbell LI, Carbajo Martinez M, Chakiachvili M. 2022.** Ensembl Genomes 2022: an expanding genome resource for non-vertebrates. *Nucleic acids research* **50**(D1): D996-D1003.
